# Eye and Periocular Skin Involvement in Herpes Zoster Infection

**Published:** 2015

**Authors:** Chris D. Kalogeropoulos, Ioannis D. Bassukas, Marilita M. Moschos, Khalid F. Tabbara

**Affiliations:** 1 Department of Ophthalmology, Ocular Inflammation Service, University of Ioannina, Ioannina, Greece; 2 Department of Skin and Venereal Diseases, University of Ioannina, Ioannina, Greece; 3 Department of Ophthalmology, University of Athens, Athens, Greece; 4 The Eye Center and the Eye Foundation for Research in Ophthalmology, Riyadh, Saudi Arabia

**Keywords:** Eye, Periocular Skin Involvement, Herpes Zoster

## Abstract

Herpes zoster ophthalmicus (HZO) is a clinical manifestation of the reactivation of latent varicella zoster virus (VZV) infection and is more common in people with diminished cell-mediated immunity. Lesions and pain correspond to the affected dermatomes, mostly in first or second trigeminal branch and progress from maculae, papules to vesicles and form pustules, and crusts. Complications are cutaneous, visceral, neurological, ocular, but the most debilitating is post-herpetic neuralgia. Herpes zoster ophthalmicus may affect all the ophthalmic structures, but most severe eye-threatening complications are panuveitis, acute retinal necrosis (ARN) and progressive outer retinal necrosis (PORN) as well. Antiviral medications remain the primary therapy, mainly useful in preventing ocular involvement when begun within 72 hours after the onset of the rash. Timely diagnosis and management of HZO are critical in limiting visual morbidity. Vaccine in adults over 60 was found to be highly effective to boost waning immunity what reduces both the burden of herpes zoster (HZ) disease and the incidence of post-herpetic neuralgia (PHN).

## INTRODUCTION

Varicella zoster virus (VZV, HHV-3) is a highly contagious DNA virus. The primary Infection typically occurs in childhood. The virus is responsible for causing two distinct diseases:

Varicella (chickenpox), the primary, highly contagious, airborne VZV infection, leads to a lifelong latent contagion of a group of sensory ganglionic neurons in the trigeminal or dorsal root of the host (1).

In contrast, herpes zoster (HZ; shingles) is a sporadic neurocutaneous disease that results from the reactivation of latent VZV (2). Varicella zoster virus infection can be reactivated either “spontaneously” or by specific triggers. Five genotypes of VZV with distinct geographical distribution have been defined using molecular techniques. Genotypes B and C are predominantly found in Europe and North America, whereas J, J2, and A1 are most prevalent in Africa and Asia (3). Different genotypes can establish latency within the same host, and also lead to independent reactivation episodes (4).

The incidence of HZ is about 3.0-3.5 per 1000 persons per year with a mean estimated lifetime attack rate of about 30% (5). HZ is rare in young people; however, its incidence increases sharply after 50 years of age. It reaches roughly ten cases per 1000 persons by the age 80, i.e. at least 50% of those surviving to 85 years of age will have had HZ (6).

Herpes zoster is more frequent in individuals with impaired cell-mediated immunity (CMI) due to disease, drugs, or radiotherapy. The incidence of herpes zoster is 20 to 100 times greater in HIV-positive patients, transplant recipients, or people with certain malignancies than in immunocompetent individuals (7). In the general population, older age is the most significant independent risk factor for HZ (8). Although HZ is not as contagious as primary varicella infection, patients can transmit VZV to non-immune contacts.

## PATHOGENESIS OF VZV INFECTIONS

Site of entry of the varicella zoster virus is the upper respiratory tract. The virus proliferates in the adjacent pharyngeal lymphoid tissue and from there spreads to the skin causing varicella. It also induces lifelong immunity (9) and latency by evading the host immune system (10). Latency is a universal biological property of all herpes viruses. Taken together, the immune response to VZV infection is quite complex:

1) After the local innate immunity barrier is overcome, the virus spreads in the body in the form of a cell-associated viremia within memory T cells (11).

2) Humoral immunity is built-up during the primary infection; however, diseases with defects in antibody synthesis are not associated with significantly increased HZ disease risk (9).

3) Cell-mediated immunity (CMI) is the main component of host’s response to VZV infection and varicella. Also, the course of HZ is more severe in patients with CMI defects (12).

Adequate T-cell immunity is essential for safeguarding latency. Chemical or physical factors, stress, insolation or solar radiation, malignant diseases, immunosuppressive treatments, or HIV infection may trigger VZV reactivation.

## CLINICAL MANIFESTATIONS AND DIAGNOSIS OF HERPES ZOSTER OPHTHALMICUS

Periocular Skin Lesions

Unilateral radicular pain and a vesicular rash (grouped vesicles on an erythematous base), usually limited to one or two adjacent dermatomes, are characteristic of HZ (13). Most frequent localizations are dermatomes T3, S3, and the first trigeminal branch that can be associated with herpes zoster ophthalmicus (HZO). Prodromal symptoms may precede the rash by several days. They include pruritus, dysesthesia, and pain. In rare instances, pain without eruption may develop (zoster sine herpete). Healing may take more than four weeks (14).

**Figure 1:**
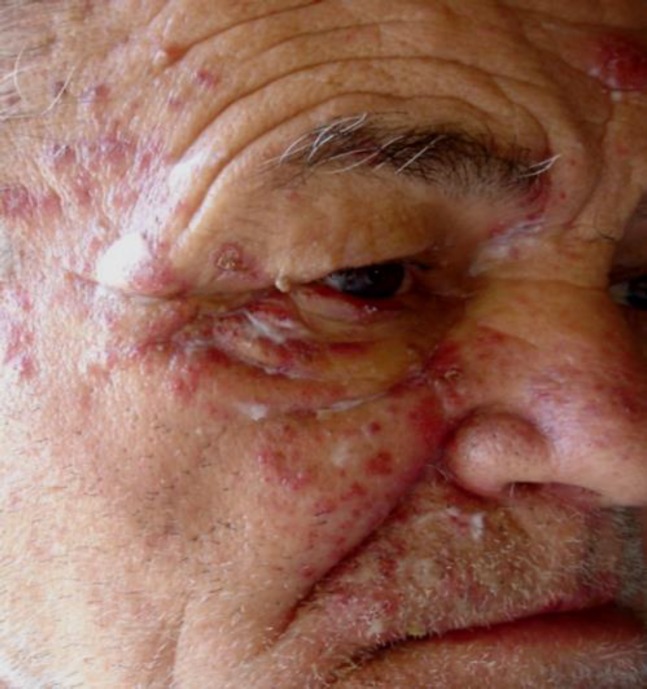
Herpes Zoster of the second Trigeminal Branch

**Figure 2:**
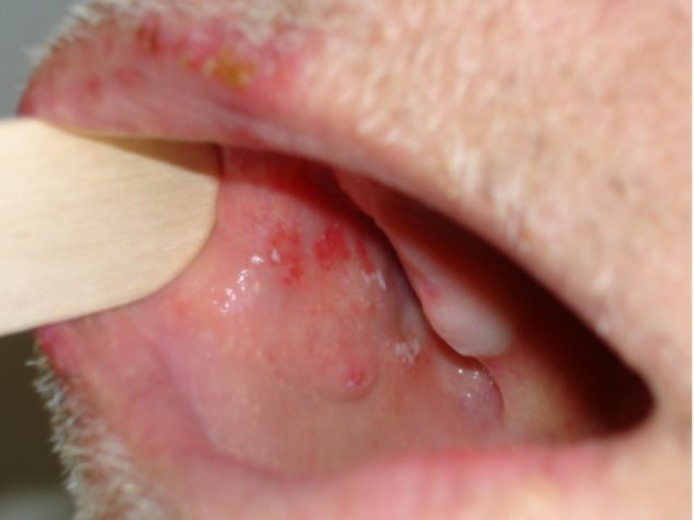
Oral Herpes Zoster Lesions

The first and second branches of the trigeminal nerve are affected in about 10% to 20% of HZ episodes (15). Correspondingly, lesions are found on the forehead, scalp, and upper eyelid (first branch) or the cheek, lower eyelid, and upper lip (second branch; Figure 1). Emergence of skin lesions on the tip of the nose is a finding characteristic for the involvement of the nervus ophthalmicus ramus nasociliaris and a sign highly predictive of HZO (75% of patients; Hutchinson’s sign II). Involvement of the second (and third) branch of the trigeminal nerve may additionally affect the mouth and manifest as ipsilateral mucosal erosions (Figure 2).

Visceral dissemination in immunocompromised patients affecting the lung, liver, and brain leads to a significant mortality rate, ranging from 5% to 15% (16).

The spectrum of the differential diagnosis of facial HZ includes herpes simplex virus (HSV) infections, impetigo, contact dermatitis, insect bites, and drug eruptions. Herpes simplex is usually more restricted by its localization; however, more disseminated or segmental-zosteriform variants of the HSV can be confused with HZ, although they never show dermatomal distribution. Moreover, HSV lesions are associated with varying recurrences patterns.

In contact dermatitis, the typical prodromal signs of HZ are absent and pruritus is the predominant symptom. To date VZV DNA detection by polymerase chain reaction (PCR) is the most useful laboratory test for diagnosis confirmation (17).

The skin of the lower eyelid is affected and slight conjunctivitis is evident. The lesional skin is covered with a fusidic acid containing crème.

Buccal mucosal erosions in a patient with herpes zoster affecting the 2nd trigeminal branch (same patient as in Figure 1).

*Ocular Involvement*

Herpes zoster with involvement of ocular structures (i.e. HZO) accounts for approximately 10% to 20% of HZ cases.

Accumulated evidence suggests that eye involvement may be an independent unfavorable prognostic factor in patients with HZ. A population-based follow-up study was conducted to assess the risk of a subsequent cancer diagnosis.

**Table 1:** Ocular Involvement

**Ocular Involvement**	
**Lid and Adnexa**	Blepharitis—secondary infection with Staphylococcus aureus Lid edema Vesicular lip eruption Phthisis bulbi Cicatricial entropion with or without trichiasis Cicatricial ectropion Chronic permanent scarring Canaliculitis Ptosis Dacryoadenitis
**Conjunctiva**	Hyperemic follicular conjunctivitis (rare) Papillary conjunctivitis Petechial hemorrhagic conjunctivitis Vesicular conjunctivitis Conjunctival edema Cicatricial conjunctival changes
**Cornea**	Acute epithelial keratitis Coarse punctate keratitis “Pseudodendritic” keratitis (“zoster dendrites”) Mucous plaques Nummular anterior stromal keratitis Interstitial keratitis Fascicular vascularizing keratitis Serpiginous ulceration Disciform keratitis Corneal hypesthesia or anesthesia Neurotrophic keratitis, with or without melting and perforation Corneal scars Calcific band keratopathy Lipid keratopathy Corneal edema Peripheral corneal ulceration Epithelial inclusion cysts
**Sclera and Episclera**	Scleritis Episcleritis
**Anterior Chamber Angle**	Trabeculitis Glaucoma, secondary to trabeculitis or attendant steroids
**Pupil**	Adie’s tonic pupil Horner’s syndrome
**Uvea**	Iritis Sectoral iris atrophy Iridocyclitis, occasionally “plastic” with hypopyon Anterior segment necrosis Choroiditis Panuveitis
**Lens**	Cataract, secondary to inflammation or attendant steroids
**Vitreous and Retina**	Retinitis or neuroretinitis Macular edema Retinal vasculitis ( Perivasculitis, arteritis and thrombophlebitis) Retinal detachment, exudative or rhegmatogenous Acute retinal necrosis (ARN) Progresive outer retinal necrosis (PORN)
**Optic Nerve**	Optic neuritis Retrobulbar neuritis Optic atrophy Papillitis and papilledema Neuroretinitis (papilledema and macular star)
**Extraocular Muscles**	Extraocular muscle palsies (ophthalmoplegia), myositis Ptosis Diplopia Exophthalmos Proptosis
**Orbit**	Orbital apex syndrome

During the 1-year follow-up, cancer was diagnosed in 4.8% of the population. Thus, HZO may be a marker of a higher risk of cancer in the subsequent year (18).

Likewise, HZO may represent a marker of increased risk of stroke during the 1-year follow-up period (19). A subsequent study found that patients previously infected with HZV have a 4.52-fold higher risk of stroke than their counterparts who had never been infected with HZV.

Most cases of HZO have a prodromal period that may include fever, malaise, headache, and eye pain. Ocular involvement (Table 1) occurs in about 50% of patients with HZV infection. 

In Africa, HZO is very frequent and severe, and in some regions of Africa, HZ is a marker for HIV infection (20, 21).

The overall visual outcome is good in HZO patients, who receive antiviral therapy. Hutchinson's sign and zoster anterior uveitis are potential predictors of visual loss. Therefore, in the presence of these predictors close monitoring is mandatory (22).

## Lids

Early in the course of the disease, the eyelids become hyperemic and edematous. If the swelling is significant, ptosis may occur. Herpes zoster often results in cicatricial skin alterations. The skin of the eyelids is relatively thin, and secondary scarring may be more apparent in this area. Conversely, later restrictions in eyelid mobility through skin scarring or through a sustaining palsy of the orbicularis oris muscle result usually in lagophthalmos.

Mild lagophthalmos or exposure of the cornea can be treated using artificial teardrops or ophthalmic ointment. When ocular lubrication alone is insufficient to treat the corneal signs and symptoms of lagophthalmos, a surgical treatment is needed that targets the anatomic abnormality causing the lagophthalmos.

## Conjunctiva

Conjunctivitis from HZO infection can induce a pseudomembranous, membranous, or follicular response. The conjunctiva is hyperemic and edematous, often with petechial hemorrhages (23). The findings usually resolve within one week. Vesicles may be present on the bulbar or palpebral conjunctiva. Topical antibiotic drops may be administered to prevent a secondary bacterial infection. On the other hand topical steroids may be used in cases that significant inflammation.

## Cornea

Approximately 65% of patients who develop HZO present with corneal involvement including punctate epithelial keratitis, early pseudodendrites, anterior stromal infiltrates, corneal mucous plaques, disciform keratitis, neurotrophic keratitis, or exposure keratitis (24).

The clinical features of corneal disease represent direct viral insult, antigen–antibody reactions, delayed cell-mediated hypersensitivity reactions, and neurotrophic damage (25).

Early lesions are likely due to direct tissue damage caused by the florid viral infection. On the other hand, the late sequelae are probably the consequences of vasculitis, immune reactions to viral antigens, or delayed hypersensitivity reactions, or may occur secondary to nerve and tissue damage. Thus, HZO-induced corneal damage may occur in the early stages of the infection, or months or even years later.

Despite appropriate medical and surgical management, significant ocular damage and loss of vision may result in addition to pain and light sensitivity.

## Epithelial Keratitis

The first corneal finding, on slit lamp examination, is punctate epithelial keratitis (24), appearing as multiple, focal, swollen lesions that stain with Rose Bengal or fluorescein dye. These lesions probably contain live virus and resolve or progress to dendrite formation. Punctate epithelial keratitis may present during the first 2 days after the initial skin rash. Dendrites typically develop later (between the 4th and 6th day after the eruption); however, they can also appear many weeks later (25). They emerge as elevated plaques due to swollen epithelial cells. Herpes zoster dendrites form branching or “medusa-like“ patterns and have tapered ends (HSV dendrites have terminal bulbs). Punctate and dendritic lesions can induce anterior stromal corneal infiltrates (26, 27).

Stromal Keratitis—Anterior Stromal Keratitis

This complication can affect as many as 25 to 30% of patients with HZO (28). Earliest findings of corneal stromal involvement become evident during the second week of the disease. The condition is characterized clinically by multiple fine granular infiltrates in the anterior corneal stroma below the epithelial layer. The infiltrates are thought to be the result of antigen-antibody interactions due to viral proliferation in the epithelium (24, 27). Anterior stromal keratitis may have a prolonged and recurrent course.

Stromal Keratitis—Deep Stromal Keratitis

Deep stromal keratitis is a comparatively late manifestation of herpes zoster eye disease. It is relatively uncommon and typically develops 3 to 4 months after the initial acute herpes zoster episode, although it has been reported to develop from 1 month to many years later (25). Deep stromal keratitis may affect all levels of the stroma, or may consist of peripheral infiltrates with a surrounding immune ring. Corneal edema is a prominent feature at this stage, usually with anterior chamber inflammation. Without corticosteroid treatment, the tissue damage may progress into a destructive, chronic inflammatory response that results in neovascularization, scarring, and corneal ulceration. In addition, lipid deposition may occur. Use of corticosteroids is imperative to interrupt the underlying inflammatory process and prevent permanent alterations.

**Figure 3:**
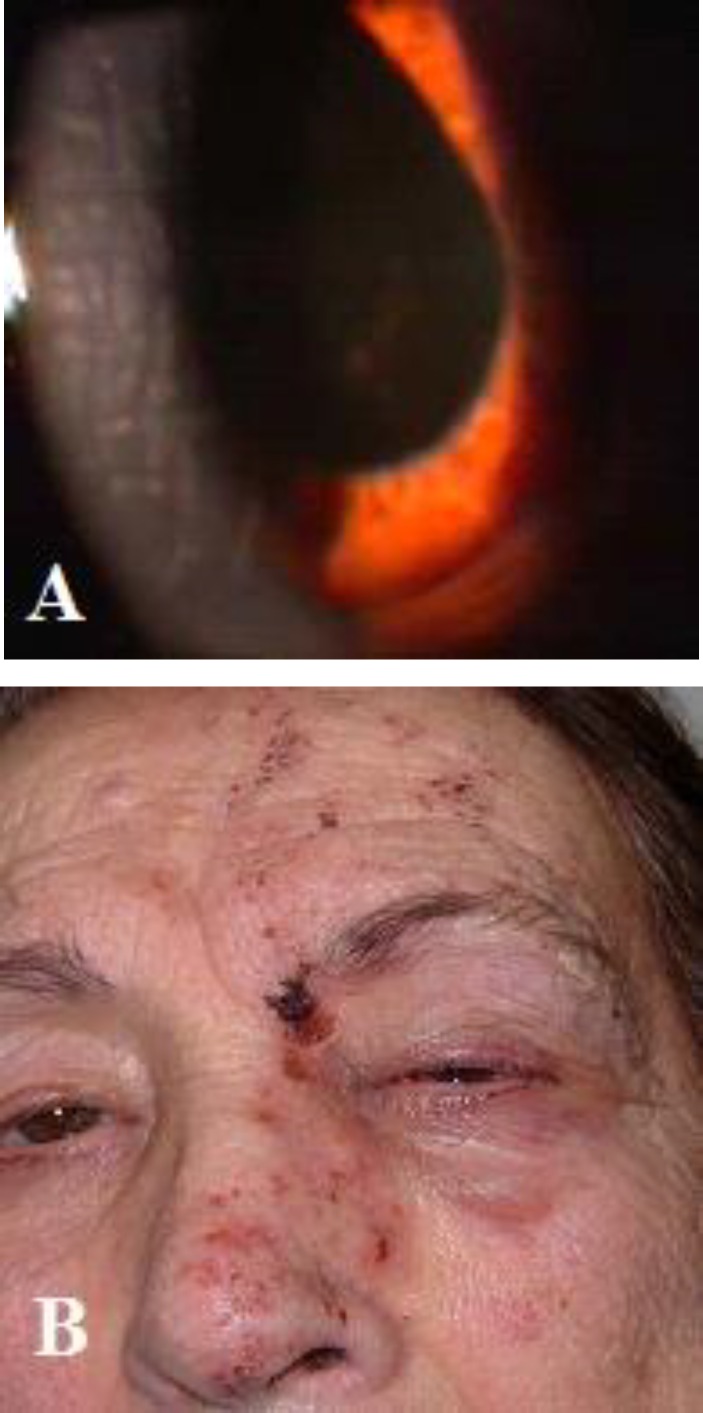
Herpes zoster ophthalmicus

The pathogenesis of stromal disease probably involves a delayed cell-mediated hypersensitivity reaction. Additionally, Reijo et al. described endotheliitis and subsequent endothelial cell loss associated with late stromal keratitis and keratouveitis (Figure 3) (29).

Some patients may develop concurrent increased intraocular pressure (IOP) (20). Increased IOP associated with anterior segment inflammation from zoster can be difficult to treat and is long lasting. The use of prostaglandin analogs to control IOP is usually avoided as they may increase the intraocular inflammation.

Neurotrophic Keratopathy and Neurotrophic Keratitis

Neurotrophic keratopathy and neurotrophic keratitis are the result of decreased corneal sensation from VZV-mediated destruction, which can cause susceptibility to mechanical trauma, decreased lacrimation, and delayed epithelial healing (25). Corneal thinning is a serious complication that may lead to corneal perforation. Because VZV infections affect sensory nerves, some patients experience a certain degree of hypoesthesia.

Decreased corneal sensation reduces blinking resulting in corneal exposure and dry eye. Surprisingly, these patients have little or no discomfort because of their hypoesthesia. At this stage, sterile corneal ulcerations may occur, but can become superinfected and give rise to a secondary bacterial infection. If such ulcerations are left untreated, the cornea becomes progressively opaque, continues to thin, and eventually become perforated.

A. keratitis (Descemet membrane folds) along with anterior uveitis (posterior synechiae remnants and irregular pupil) suggesting keratouveitis, and B. skin lesions corresponding to the affected dermatomes.

Peripheral Corneal Ulcers

Peripheral corneal ulcers are a rare complication associated with VZV corneal infections. The ulcers may resemble Mooren’s ulcers and are typically associated with anterior uveitis or stromal keratitis, but regional exposure and neurotrophic keratitis must also be considered.

Corneal Scars

Corneal scarring commonly occurs after a VZV corneal infection. The corneal scar is either a faint stromal haze or an opaque lesion of the cornea along with corneal thinning.

## Sclera and Episclera

Episcleritis and Scleritis

Herpes zoster episcleritis is localized or diffuse. Scleritis is a more serious entity. Localized stromal keratitis may accompany both conditions.

## Uvea and Retina

Uveitis

It rarely occurs with the waning of varicella and occurs more frequently with ophthalmic zoster. Anterior uveitis is often unilateral acute hypertensive plastic or granulomatous, synechiae associated with sectorial iris atrophy (30). Bilateral involvement can also be present (31).

Anterior uveitis may be associated with keratitis. The inflammation is usually mild and transient, but it frequently causes a hypertensive uveitis (elevation of intraocular pressure). Zoster uveitis can result in iris atrophy and an irregular pupil. The course of the disease may be prolonged, especially without the appropriate treatment. Herpes zoster uveitis may cause glaucoma. The total incidence of secondary glaucoma is approximately 13.1%. Most of the patients respond to antiviral and antiglaucomatous therapy, and trabeculectomy with mitomycin C is rarely performed. Uveitic glaucoma is a frequent complication of viral uveitis.

Cataract formation is also typical in HZ uveitis. Chronic inflammation can lead to endothelial cell damage, resulting in corneal edema (Table 2).

ARN and PORN Syndromes

Herpes zoster virus is considered the cause in most cases of ARN (along with HSV1, and in some rare cases, with HSV2) and PORN syndromes. Compared with ARN, PORN is a more severe viral retinitis observed in immunocompromised persons, often in those with acquired immunodeficiency syndrome.

Herpes zoster viruses can cause a broad spectrum of clinical manifestations ranging from severe ARN to slow-progressing necrotizing and non-necrotizing types of inflammation. Concerning non-ARN variants which are underdiagnosed, patients could potentially benefit from earlier recognition and treatment (32).

**Table 2:** Differential patterns of herpetic keratouveitis and herpetic anterior uveitis (HSV and VZV) Adapted from: David BenEzra, Shigeaki Ohno, Antonio G. Secchi, Jorge L. Alio, Martin Danitz Anterior segment intraocular inflammation guidelines (IOIS), 2000

**Location of Inflammation**	**Herpetic Keratouveitis**	**Herpetic Uveitis**
**Corneal inflammation**	Active disciform (or stromal keratitis	Rare, inactive
**Corneal hyposensitivity**	Frequent	Rare
**Iris involvement **	Uncommon	Vasculitis, Sectoral atrophy
**Iridoplegia**	Uncommon	Frequent
**Keratic precipitates**	Granulomatous or nongranulomatous Associated with keratitis	Granulomatous Scattered
**Cells in anterior chamber**	± or +	++ or +++
**Eye pressure**	Standard or high	High or very high
**Unilateral** *	Always	Always
**Course**	Chronic / recurrences	Acute / recurrences

* The activity of uveitis and keratouveitis is always unilateral but corneal and iris scars can infrequently be bilateral.

**Figure 4:**
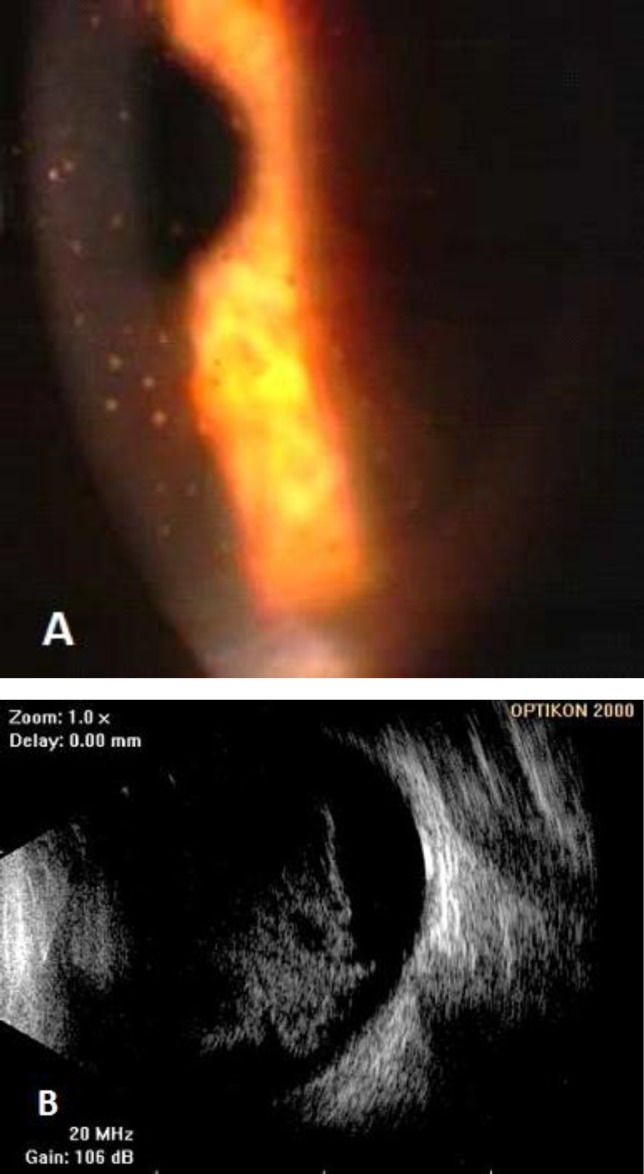
Unilateral HZV panuveitis

**Figure 5:**
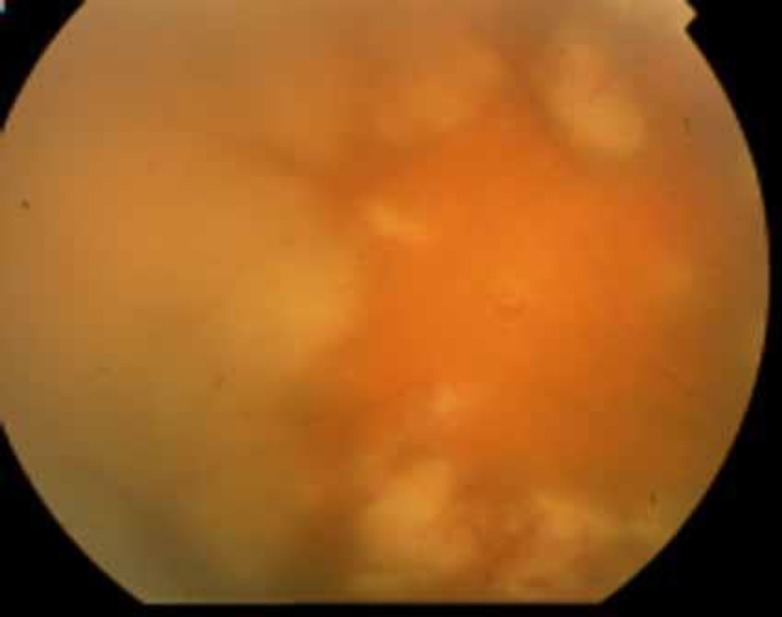
Acute retinal necrosis due to HZV (detected by PCR in aqueous humor) with the characteristic confluent necrotic zones.

All patients with ARN have necrotic retinal lesions that progress quickly, whereas patients in the non-ARN group have necrotic retinal lesions that progress slowly. Necrotizing variants can be also noted as slowly progressing lesions. Some of the patients in the non-ARN group have posterior uveitis without retinal lesions; thus, vitritis, vasculitis, papillitis, and panuveitis are hallmarks of these cases (Figure 4), without any distinct features (32). Rapidly coalescent peripheral patches of retinal necrosis, occlusive vasculitis, and vitreous inflammation characterize ARN (Figure 5). Currently accepted diagnostic criteria for this condition are compiled in Table 3. Progressive outer retinal necrosis (PORN) is a morphologic variant of acute necrotizing herpetic retinitis. PORN occurs most often in patients with advanced AIDS or patients who are otherwise profoundly immune-suppressed.

A. keratic precipites and B dense vitreous inflammation (B-mode).

The most common cause of PORN is VZV; HSV has also been isolated from PORN lesions. As with ARN, the retinitis also initiates with patches of outer retinal whitening with rapid merging. In contrast to ARN, the posterior pole may be recruited early, vitreous inflammatory cells are typically absent, and the retinal vessels are, at least initially, minimally affected. The visual prognosis is poor; in the largest series reported to date, 67% of patients had a final visual acuity (VA) of no light perception. The disease is often resistant to intravenous acyclovir alone. Successful management has been reported with a combination of systemic acyclovir and intraocular therapies with foscarnet and ganciclovir.

**Table 3:** American Uveitis Society Criteria for Diagnosis of ARN Syndrome (33).

**One or more foci of retinal necrosis with discrete borders, located in the peripheral retina** *
**Rapid progression in the absence of antiviral therapy**
**Circumferential spread**
**Occlusive vasculopathy with arteriolar involvement**
**Prominent vitritis, anterior chamber inflammation**
**Optic neuropathy/atrophy, scleritis, pain supportive but not required **

* Macular lesions do not exclude the diagnosis in the presence of peripheral retinitis. Am J Ophthamol. 1994; 117:663-667 (33)

Multifocal Posterior Necrotizing Retinitis

Most patients whose eyes are affected demonstrate the known peripheral retinitis pattern, even though that pattern affects mainly the posterior pole in some patients. Most of those lesions are peripherally localized, focally distributed retinal necroses. They are usually well demarcated, with rapid circumferential progression and rare involvement of the posterior pole. Patients with the involvement of the posterior retina seem to have an unfavorable eyesight outcome during the first 2 years (34). The “Cox proportional hazards model” suggests a higher incidence of retinal detachment in patients with confluence of multifocal lesions of posterior necrotizing retinitis (34). Both conditions commonly cause retinal detachment. The prognosis is poor in patients with PORN; most patients ultimately have no light perception vision (35). The visual prognosis in patients with ARN is better, with many patients achieving a VA of 20/40 (36). A number of necrotizing retinopathies, including peripheral toxoplasma retinitis, syphilis, Behçet’s disease, intraocular lymphoma, and aspergillosis, simulate ARN (37). Since the vast majority of the patients have positive serologies for herpesviruses and Toxoplasma gondii, laboratory investigations may not be helpful in making proper etiological diagnoses. Aqueous humor analysis by PCR (38) and a Witmer-Goldmann coefficient determination are helpful for diagnosis and disease management. Bilateral involvement is observed with both clinical forms in about one-third of patients but may affect as many as 80% of patients with untreated disease (39). Treatment includes extended administration of intravenous and oral acyclovir and corticosteroids. Non-necrotizing herpetic retinitis (non-necrotizing posterior uveitis) may occur in patients with herpetic infections. In 13% of cases estimated as “idiopathic posterior uveitis”, PCR-based assays and local antibody analysis of aqueous fluid samples for herpesviruses, confirmed a viral etiology. Inflammation is typically bilateral, with cystoid macular edema (CME), simulating a birdshot-like retinochoroidopathy or a vascular occlusive bilateral retinitis.

COMPLICATIONS OF HZV

**Figure 6:**
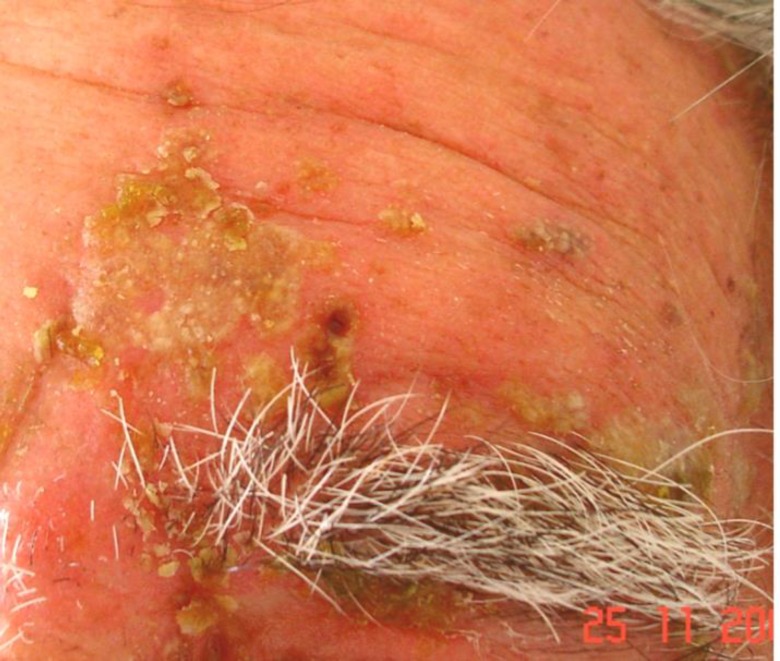
Herpes zoster ophthalmicus with secondary bacterial superinfection (impetiginization). Honey-colored crusts on erythematous base at the sites of zoster skin lesions and conjunctivitis. Plentiful Staphylococcus aureus was grown from the lesions.

The complications of HZV can be divided into four groups (cutaneous, visceral, neurological, and ocular). The incidence of all complications increases with age (40). Ocular involvement was described above. Secondary bacterial superinfection by Staphylococcus aureus or Streptococcus pyogenes (Figure 6) is the most frequent cutaneous complication of HZV and may affect the development of subsequent post-herpetic neuralgia (PHN). PHN is defined as pain lasting after the rash has disappeared, usually when pain is present for 90 days after the onset of rash (13). Except for PHN, neurologic complications of HZO are rather rare and may include acute or chronic encephalitis, myelitis, aseptic meningitis, autonomic dysfunction, motor neuropathies, and cranial nerve palsies (41).

About 20% of HZ patients develop PHN. Similar to the pain intensity during overt HZ, patients with HZ affecting dermatomes T3, S3, and the first trigeminus branch (HZO) are most prone to the subsequent development of PHN. Post-herpetic neuralgia may persist for a lifetime and has a significant effect on quality of life and use of the healthcare system’s resources. The risk of development and duration of PHN rise with age, particularly beyond the sixth decade (42). Herpes zoster ophthalmicus is established as an independent risk factor for PHN development (9). While the pathophysiology of PHN remains unclear, it likely results from VZV-related damage of the sensory ganglion and may reflect continued inflammation by enhanced production of VZV (43).

## Treatment of Complications

Herpes zoster is painful and associated with diminished quality of life (44). The treatment of HZV has three primary milestones:

1) Treatment of the acute viral infection

2) Treatment of the acute pain associated with HZV

3) Prevention of PHN and other complications (45).

Early commencement of antiviral therapy is essential (46). Nevertheless, current International Herpes Management Forum (IHMFR) Guidelines (47) recommend that all patients with HZO presenting within one week of rash onset should receive antiviral therapy in the doses indicated above.

Antiviral drugs are highly effective in controlling the severity and duration of HZ; however, they do not reliably prevent the development of PHN with overall mild adverse effects such as headache, nausea, and gastrointestinal disturbance. Three oral nucleoside analogs are approved worldwide for the treatment of HZ. The older acyclovir [800 mg every 4 hours for 7 days for immunocompetent adults] had been the standard of care for treating cutaneous HZ. Famciclovir [500 mg every 8 hours] and valacyclovir [1000 mg every 8 hours] are now preferred because of their superior pharmacokinetic characteristics and simpler dosing regimens. The recommended duration of treatment for immunocompetent adults is 7 days. Brivudin [125 mg, once daily p.o.] is available in some countries but its use is limited to immunocompetent patients because of a potentially fatal interaction with 5-fluorouracil (5-FU) (45). Guidelines suggest initiation of antiviral therapy within 72 hours of rash onset, although there are no data showing that later initiation is not helpful. Corticosteroids (20-60 mg/d prednisone equivalent) may help control pain in acute HZ. However, their random use is limited due the high prevalence of comorbidities, such as glaucoma, among individuals at risk for HZ development (older adults) (45). Individualized analgesic treatment schedules according to pain severity are preferred, and include tricyclic antidepressants, gabapentin, pregabalin, opioids, topical lidocaine, or combinations of these medications. For World Health Organization (WHO) grade 1 pain, treatment with nonsteroidal antiphlogistics is preferred (paracetamol 3-4 x 500 mg/d p.o.; indomethacin 50-150 mg/d; ibuprofen 2 x 800 mg/d p.o.; acetylsalicylic acid 3-4 x 0.5-1 g/d p.o.; naproxen 2 x 500 mg/d p.o.). Patients with WHO grade 2 pain, which is common in HZO, may require low-potency opioids such as tramadol (2-3 x 50-100 mg/d) alone or in combination with amitriptyline (20-50 mg/d p.o.), gabapentin (900-2400 mg/d), or clonazepam (1.0-2.0 mg/d). α2δ ligands such as gabapentin and pregabalin have been proven to effectively control PHN and improve the quality of life in large placebo-controlled trials (45). Finally, for patients presenting with WHO grade 3 pain, high-potency opioids such as morphine or fentanyl with individualized dosing regimens are recommended in conjunction with “co-analgesics” as required. 

Treatment of PHN is imperative, especially for elderly patients with HZO. These elderly patients are highly sensitive to short periods of compromised health, which can have a long-term impact on daily activities (48). Antiviral treatment of acute HZ and the use of systemic corticosteroids to diminish the degree of PHN are usually sufficient. It is worth noting that various treatment strategies are offered without adequate consensus about their effectiveness (49). However, recent meta-analyses found insufficient evidence to generally recommend these practices for all PHN patients (50,51). 

Finally, HZ in pregnancy is an alarming, but not detrimental, event. If indicated, intravenous acyclovir can be given. Except in cases of generalized HZ, no substantial viremia occurs and there are no additional risks for the mother or child.

## TREATMENT OF HZO

The general framework of therapeutic measures for HZ also applies in the case of HZO. Patients with uncomplicated HZO can be adequately treated with oral acyclovir (800 mg, five to six times daily) for 7 to 10 days. Studies report alleviation of pain with oral acyclovir during the initial stages of the disease. This effect is especially notable if the treatment is initiated within the first 3 symptomatic days, and it may have a favorable effect even though this effect on PHN is controversial (52-54). Additionally, acyclovir administration in first 72 hours accelerates the resolution of skin lesions and decreases the incidence of dendritic and stromal keratitis as well as anterior uveitis (55,56). Valacyclovir has higher bioavailability and has been shown to be equally safe and effective for the treatment of HZ at a dosage of 3000 mg per day (57); if administered on a 7-day schedule, it prevents ocular complications of HZO (58). Famciclovir, 500 mg orally three times a day for 7 days, may also be used (59). Intravenous acyclovir is recommended in immunocompromised patients and in immunocompetent with ARN or severe non-necrotizing posterior uveitis or panuveitis. (60,61).

**Table 4:** Schematically overviewed HZ treatment

**Disorder**	**Treatment**
**Blepharitis/conjunctivitis**	Palliative care with cold compresses, topical lubrication and a topical broad spectrum antibiotic for the prevention of secondary bacterial infections (usually Staphylococcus aureus and secondarily Streptococcus pyogenes)
**Epithelial keratitis**	Debridement (optional) and topical lubrication
**Stromal keratitis**	Topical steroids/ The beneficial effect of topical acyclovir is unproven
**Neurotrophic keratitis**	Topical lubrication, topical antibiotics for secondary infections, tissue adhesives and protective contact lenses to prevent corneal perforation and topical or oral steroids to alleviate inflammation
**Scleritis/episcleritis**	Topical nonsteroidal anti-inflammatory agents and/or steroids along with topical and oral acyclovir for a long-term period (especially for scleritis)
**Uveitis**	Oral acyclovir for at least six months (usually for a year) and topical steroids in tapering doses
**ARN/ PORN**	Intravenous acyclovir (1,500 mg per m2 per day divided into three doses) for 10 to 21 days, followed by oral acyclovir (800 mg orally three to five times daily) for 14 weeks or more

Patients with severe and progressive disease despite acyclovir therapy may be treated with ganciclovir or foscarnet. Intravitreal injections of 2 mg/0.1 ml ganciclovir may be useful as adjunctive therapy.

In immunocompetent patients with ARN oral steroids administered in tapering doses (based on the amount of vitritis) after the patient has received intravenous acyclovir for 48 hours could be helpful in terms of reduction of inflammation.

Laser/surgical intervention may be performed as required.

*Surgical Treatment of HZO complications*

When ocular lubrication alone is insufficient to treat the corneal complications of lagophthalmos, surgical treatment should be planned.

In some cases, orbicularis oculi muscle palsy can be corrected by mechanical means. Both surgically implanted springs and gold weights have been used to achieve eyelid closure. Elevation of the palsied lower lid may also be necessary to achieve adequate treatment of the lagophthalmos (23). Tarsorrhaphy can become an additional technique to correct lagophthalmos. Botulinum-A toxin injected into the upper eyelid produces temporary ptosis and protection of ocular surface (62).

As the corneal epithelium deteriorates, a punctate keratopathy can be seen. If the epithelial breakdown progresses, significant corneal epithelial defects may occur. Corneal ulcerations develop that are sterile in nature but can become infected. If left untreated, the cornea may become opaque, continues to thin, and eventually may perforate. Several studies have questioned the use of amniotic membrane (AM) grafts for the treatment of severe neurotrophic corneal ulcers following HZO. Authors of these studies achieved rapid re-epithelization and healing with reduced inflammation (63,64).

Small corneal perforations of up to approximately 1.5-mm diameter can be treated emergently with off-label use of cyanoacrylate glue or fibrin-based tissue adhesives (65-67). The purpose of using these materials is to allow the cornea to heal the small defect. If the defect is larger than what can be safely treated with a tissue adhesive, a patch graft (conjunctival) to the cornea is needed. Interrupted sutures should be used to avoid total loss of the graft’s sutures if one single region thins and the sutures become loose, break, or cheese wire through the tissue. Single-layer and multilayer AM grafts have been successfully used to close corneal perforations as large as 1.5 to 2.0 mm. These materials have been used with fibrin tissue adhesive to seal the defect or with sutured AM alone (68-70). Investigators demonstrated an increased rate of corneal re-epithelization and reduced rate of corneal melts with AM transplants (63, 64,71).

In peripheral corneal ulcers, a conjunctival graft can be used to stabilize the cornea and halt the progression of corneal thinning. To accomplish a successful graft, the surface of the ulcer must be cleaned of any necrotic debris and the corneal epithelium removed from the region of the planned conjunctival graft site. Use of an AM may provide protection to the conjunctival graft and may reduce localized inflammation and neovascularization. In corneal scars if the residual corneal thickness, posterior to the scar, is <250 μm and the corneal endothelium is compromised, a penetrating keratoplasty (PK) will be necessary. If, however, the corneal endothelium is normal, then a tectonic lamellar keratoplasty can be performed to restore corneal clarity.

Corneal scars in the anterior half of the cornea can be treated by keratectomy or lamellar keratoplasty. If the patient develops increased IOP and anterior segment inflammation, uveitic cataracts and glaucoma commonly occur. Therefore, cataract surgery, and in some cases, glaucoma filtering surgery, are required. In ARN patients who at baseline are in a state of relative immunosuppression, the outcomes obtained are good, suggesting a role for strong immune reactions in the development of retinal detachment in ARN.

A 3600 laser photocoagulation can be performed to “secure” the high-risk zones (the confluent areas of retinal necrosis in the peripheral retina).

Rhegmatogenous retinal detachment, which has a high-risk of occurring (43%) (72), requires vitreous surgery with silicone oil tamponade. Successful retinal reattachment can be attained, although in some cases silicone oil cannot be removed due to high-risk for recurrent detachment. Prophylactic vitreous surgery can also be performed (21%) in eyes with no retinal detachment (72). The indication for surgery in these eyes is an acute worsening of vitreous haze obscuring the view of the fundus.

## PREVENTION/HZ VACCINATION

Live attenuated herpes zoster vaccine ((Zostavax®, Merck) can prevent herpes zoster ophthalmicus. In elderly immunocompetent persons, the recurrence rate is estimated to be less than 1% per year (73).

The use of HZ vaccine has been reported to be safe in patients with a history of HZ (74). The efficacy of the vaccine, however, has been shown to wane over the first 5 years after vaccination (75). It is interesting to note that the safety and efficacy of HZ vaccine in persons 80 years of age or older showed no significant difference from that in younger individuals (76). In the shingles prevention study, the efficacy of HZ vaccine in preventing PHN did not show a reduction in persons 70 years of age or older (77).

The varicella-zoster vaccine is available in a formulation that contains a minimum of 19,400 plaque forming units per dose. The vaccine was developed for the protection against the onset of varicella-zoster infections.

The varicella-zoster vaccine was evaluated in a large efficacy study known as “the Shingles Prevention Study Group.” The study admitted subjects 60 years of age or older with a follow-up period of 3 years post-vaccination.(76) The study revealed that the incidence of herpes zoster was 51% lower in the group of patients who received the vaccine 5-4 cases per 1000 person-years compared to 11.1 cases per 1000 person-years among the control group (p<0.001).

Notably, the mean duration of pain among subjects who received the vaccine was also significantly shorter compared to those who received placebo.

Other available herpes zoster vaccines, such as the Varivax® and the ProQuad® (both Merck) contain significantly lower titers of live attenuated virus and are not recommended for herpes zoster (and HZO) prophylaxis. In older individuals, these are of insufficient potency to elicit an increase in cell-mediated immunity,

Antiviral Agents to Prevent Complications of HZO (Guidelines)

Antiviral therapy is most beneficial in persons who develop ocular complications of Herpes zoster or who are at risk of complications from HZO, including older individuals and immunocompromised patients. The use of antiviral agents within the first 3 days after skin rash may decrease the severity of HZO complications. The most commonly used antivirals include acyclovir, valacyclovir, and famciclovir. Valacyclovir or famciclovir is preferred over acyclovir because of the reduced frequency of dosing and higher serum levels. A group of experts developed recommendations for the management of HZ. Harpaz et al. reported on the prevention of HZ by the Advisory Committee on Immunization Practices (ACIP) (78).

To prevent the HZO complications, patient is given acyclovir 800 mg orally five times daily. Alternative antivirals include valacyclovir or famciclovir to be administered within the first 72 hours after the skin rash for a period of 7 days. Valacyclovir is given at a dosage of 1 gram orally three times daily for 7 days, while famciclovir is given 500 mg orally three times a day for 7 days. Both regimens have shown reduced time to new skin lesions formation, decrease in the number of vesicles, and full crusting and cessation of pain (79).

In immunocompromised patients or patients with severe neurologic complications, acyclovir 10 mg/kg i.v. should be given every 8 hours for 7 days. Alternatively, foscarnet (in cases of acyclovir-resistance) can be used in a dose of 30 mg/kg i.v. every 8 hours until lesions are healed. Antiviral therapy for herpes zoster is recommended for all patients who are at risk of HZO, and for all immunocompromised patients and all patients aged >50 years.

Antiviral agents can help in the resolution of herpes zoster lesions and decrease the complications of HZO and the severity of PHN. Herpes zoster vaccine is recommended by the ACIP for persons 60 years or older and is used for patients with or without a history of HZ (78). The earlier the antiviral therapy is initiated the higher the likelihood of clinical response, and consequently, of significant modification of the severity of complications. It is, therefore, recommended that antiviral therapy be initiated within 72 hours after the onset of the symptoms. Nevertheless, if the patient presents after 3 days, antiviral agents can still be given.

Several studies have shown that antiviral agents may reduce the severity and duration of pain but do not reliably decrease PHN risk. Chronic neuropathic pain may develop in spite of antiviral therapy.

The only effective treatment for the prevention of HZ is the administration of the vaccine in childhood for the prevention of chickenpox. Antiviral therapy is given to all patients presenting with HZO primarily to prevent potential sight-threatening complications. Patients with uveitis should be given adjunctive therapy with corticosteroids. The potential for severe pain during HZO should not be underestimated and potent analgesics must be applied as needed. Finally, PHN may be severe and early onset of combination therapy consisting of antiviral agents and pain medications including, oral prednisone, tricyclic antidepressants, anticonvulsants, or lidocaine patches (5%) may influence disease severity and the patient’s condition.

## CONCLUSION

HZO is a serious cause of morbidity and loss of vision in the elderly population. Herpes zoster vaccination for patients with or without a history of herpes zoster may decrease the severity and ocular complications of HZO. Early detection and prompt treatment of patients who develop HZO are mandatory to prevent the serious complications of this common condition.
